# Bibliometric mapping of the landscape and structure of nutrition and depression research: visualization analysis

**DOI:** 10.1186/s41043-023-00378-2

**Published:** 2023-04-15

**Authors:** Sa’ed H. Zyoud, Muna Shakhshir, Amani S. Abushanab, Amer Koni, Moyad Shahwan, Ammar A. Jairoun, Samah W. Al-Jabi

**Affiliations:** 1 Poison Control and Drug Information Center (PCDIC), College of Medicine and Health Sciences, An-Najah National University, Nablus, 44839 Palestine; 2 Department of Clinical and Community Pharmacy, College of Medicine and Health Sciences, An-Najah National University, Nablus, 44839 Palestine; 3 Clinical Research Centre, An-Najah National University Hospital, Nablus, 44839 Palestine; 4 Department of Nutrition, An-Najah National University Hospital, Nablus, 44839 Palestine; 5 Division of Clinical Pharmacy, Hematology and Oncology Pharmacy Department, An-Najah National University Hospital, Nablus, 44839 Palestine; 6 College of Pharmacy and Health Sciences, Ajman University, Ajman, United Arab Emirates; 7 Health and Safety Department, Dubai Municipality, Dubai, United Arab Emirates

**Keywords:** Nutrition, Dietary, Bibliometric, Depression, Scopus, VOSviewer

## Abstract

**Background:**

Numerous epidemiological studies have examined the relationship between dietary intake of specific foods or nutrients and the incidence of depression and have noted that nutrition has a significant impact on mental health. Therefore, the purpose of this study is to assess the state of research, the frontiers of research, and development trends in the field of nutrition and depression using bibliometric and visual analysis.

**Methods:**

We collected publications on the topic of nutrition and depression from Scopus between 2002 and 2021. Subsequently, we utilized VOSviewer 1.6.18 and Microsoft Excel 2013 to perform bibliometric analysis and visualization. Bibliometric analysis involves retrieving documents from a singular database, such as SciVerse Scopus or Web of Knowledge, and subjecting them to quantitative and qualitative analysis. Notably, gray literature is not considered in bibliometric analysis.

**Results:**

A total of 2171 publications on nutrition and depression were found between 2002 and 2021, namely 1855 (85.44%) original articles, 190 (8.75%) reviews, 38 (1.75%) letters, and 88 (4.05%) other types of publications. The most productive country was found to be the USA (*n* = 726; 33.44%), followed by Australia (*n* = 172; 7.92%), the United Kingdom (*n* = 158; 7.28%), China (*n* = 132; 6.08%), and Canada (*n* = 131; 6.03%). The remaining publications were from other countries (*n* = 852; 39.25%). According to the citation analysis, the retrieved papers were cited on an average of 26.6 times and had an *h*-index of 105 with 57,781 citations. The most frequent terms on the map include those related to (a) fatty acid links to depression and brain inflammation, (b) depression and eating disorders, and finally, (c) adherence to the Mediterranean diet and risk of depression.

**Conclusions:**

The current study was the first novel bibliometric analysis of nutrition and depression research that used data extracted from Scopus for visualization network mapping. In recent years, the theme "Mediterranean diet adherence and risk of depression" has been identified more frequently, indicating that studies in this field have garnered considerable attention and reflect the most recent scientific advances. Researchers should continue to investigate nutrition and depression, and we believe this study provides significant information for researchers, nutritionists, and clinicians.

## Background

Depression is a chronic mental health condition associated with poor productivity and a decreased quality of life. It is considered a major cause of disability and has become a worldwide public health problem [1]. It was estimated that more than 300 million of the global population was affected by this disorder, especially young adults and adolescents, and the number of patients who suffered increased by almost 50% [1–3]. Major depressive disorder is one of the most significant contributors to the global disease burden, as it often results in debilitating disabilities for patients. Additionally, the economic burden of managing this condition through healthcare services is substantial, costing countries billions of dollars [2, 4]. Furthermore, depression is the second leading cause of death among young people and adolescents, largely due to the high prevalence of suicidal tendencies in those with the disorder [5].

Given that the available treatment options using medications are only moderately effective, with high percentages of patients experiencing relapse even with treatment, it is important to prevent the onset of depression through public health intervention [6]. While various factors can contribute to depression, lifestyle and psychological factors are known to have a direct impact. For instance, leading a sedentary lifestyle, lacking social support, or experiencing significant life stressors can increase the risk of developing depression. Similarly, individuals with a history of trauma, low self-esteem, or negative thought patterns may be more susceptible to the condition [7, 8]. Considerable evidence suggests that diet could be used as a treatment, reduce the risk of depression, and act as a protective factor against depressive symptoms [9–11].

Several studies have investigated the relationship between a healthy diet and individual nutrients and their impact on depression, such as fatty acids, vitamins, and minerals [12–14]. Moreover, intervention studies using supplements that include several nutrients, such as multivitamins, have examined the overall effect on patient mood [15–17]. In particular, increased fruit and vegetable intake has been widely associated with a better psychological and health status and a decrease in the likelihood of depressive symptoms [9–11, 18], while other studies revealed that the association is insignificant [19–21].

However, recent research has highlighted the connection between the gut microbiota and the brain, emphasizing how it affects brain function, mood, and behavior. This opens up another way nutrition might affect mental health [22–24]. In addition, polyunsaturated fatty acids (PUFAs) were also examined in several studies and revealed that PUFAs might have a beneficial effect on the prevention and/or treatment of depression through their inhibitory anti-inflammatory effect, especially since patients with depression have elevated levels of inflammation [25].

An additional dietary factor called homocysteine has been investigated in relation to depression. The findings indicated a connection between depression and low levels of folate or B12, as well as high homocysteine concentrations [26, 27]. Furthermore, antidepressant supplementation with vitamin B12 and/or folate significantly reduces the risk of relapse after successful remission [28]. Therefore, a lower risk of depression may be associated with a high intake of vegetables, fruits, whole grains, and fish [20].

Numerous dietary patterns and depression publications have been published due to the above issues. Researchers must, however, spend a significant amount of time reading and comprehending pertinent work in related areas because of the vast amount of information on this subject. Therefore, it is crucial to categorize substantial and evocative evidence to aid scientific research [29]. Although many reviews and meta-analyses can provide helpful data and reliable evidence-based medical conclusions, these techniques frequently fail to provide a comprehensive and integrated perspective for a particular field of research. Using bibliometric techniques, our research teams and other researchers have examined publication trends and research hotspots in the area of nutrition, including child nutrition [30], sports nutrition [31], nutrition and cancer [32], nutrition and dietetics [33], nutrition and COVID-19 [34], nutrition and microbiota [35], diets and breast cancer [36], dietary therapies for epilepsy [37], dietary factors of metabolic syndrome[38], Mediterranean diet [39], diet-related eHealth and mHealth [40], and HIV/AIDS and nutrition [41]. To the best of our knowledge, no prior bibliometric research has examined the most relevant scientific research on nutrition and depression.

By using statistical and mathematical techniques, bibliometric analysis refers to the quantitative and qualitative analysis of the literature on a certain topic. Many studies have been carried out in the last ten years to investigate bibliometrics in different scientific fields [42–44]. Bibliometric analysis seeks to offer a thorough overview of the body of literature on a certain topic, as opposed to systematic reviews, which aim to respond to a specific research issue based on a small number of publications [45]. It differs similarly from scoping reviews, which concentrate on defining the type and extent of research evidence [46]. Bibliometric analysis is still a useful method for acquiring an overview of national and international contributions to the literature on a given topic and for identifying research gaps that may be filled by future studies, notwithstanding these variations [43, 44]. Therefore, through bibliometrics, the authors of this study intend to compile a complete summary of all previous publications on nutrition and depression conducted over the course of the past 20 years. Their ultimate goal is to perform knowledge mapping to investigate whether there are any new frontiers or hotspots in this particular area of research.

## Methods

### Database

The quantitative and qualitative analysis of documents retrieved from a single database is bibliometric analysis. The selection of a database is crucial, and SciVerse Scopus is the database of choice for researchers [47–49]. This investigation successfully utilized Scopus to retrieve publications on nutrition and depression. Scopus, created by Elsevier, has a number of advantages over other databases such as Web of Science, Medline, and Google Scholar [50–53]: (1) Scopus contains a vast amount of research literature from a variety of disciplines and sources, such as peer-reviewed journals, conference proceedings, book series, trade publications, and patents. This publication's extensive scope makes it ideal for analyzing research trends in a variety of disciplines. (2) Scopus uses a rigorous selection process for the sources it includes; as a result, the data are generally regarded as of high quality. Furthermore, Scopus employs a team of content specialists who monitor the quality of the data and perform regular updates to ensure its accuracy. (3) Scopus allows users to conduct complex searches by author, publication, affiliation, keyword, and citation. (4) Scopus provides citation metrics such as citation counts, h-index, and cocitation analysis, which are widely used in bibliometric studies to assess the impact and influence of research publications and authors. (5) Scopus can be integrated with other tools and software, such as data visualization tools and research analytics platforms, which makes it simple to analyze and present bibliometric data in a meaningful manner. (6) Scopus includes most of the journals indexed in Web of Science, MEDLINE, and Embase, making it a comprehensive bibliometric analysis resource. All data were downloaded from Scopus on August 5, 2022, to avoid any potential bias resulting from Scopus's continual database updates.

### Search strategy

The search strategy was developed based on an extensive review of the systematic and meta-analysis literature on the topic [20, 54–57]. The research strategy was based on three steps.

1. In the first step, the following terms and expressions were employed in the title search: TITLE (nutrit*) OR TITLE (diet*) OR TITLE (eat*) OR TITLE (feeding) OR TITLE (food).
2. The second step had phrases on depression. It was applied as follows in the title search: (TITLE (depress*) OR TITLE ("seasonal affective") OR TITLE (dysthym*) OR TITLE ("affective disorder") OR TITLE ("mood disorder*") OR TITLE ("bipolar disorder")).
3. In the third step, the exclusions and limitations were applied to combine and filter the initial two steps. The study period was restricted to 2002–2021, and no language limitations were enforced. The research approach utilized the asterisk (*) as a wildcard and quotation marks ("") to narrow the search to specific terms or phrases. Errata and retracted documents were not included in the analyses.

As a direct consequence of this search strategy, a search for the title was carried out using keywords rather than a search for the title and abstract together. It is a reliable method because the search for the title will only produce a tiny number of documents that have a false-positive result [58–62]. On the other hand, a search of titles and abstracts will provide numerous false positives because the primary focus of these studies is not on diet and depression per se but rather on other issues.

### Validation of the search strategy

After refining the search query, the authors took precautions to eliminate any false positives. To accomplish this, the top 100 most-cited publications were analyzed to determine if they were relevant to the searched topic. The titles and abstracts of the most-cited documents were reviewed by two bibliometric experts to confirm the absence of false positives. After confirming that there were no false-positive results, the search query was deemed complete. In addition, the author implemented a test of correlation between the information retrieved by the search query and the actual findings for the ten most active researchers in this field. To ensure that there were no false-negative results, this was performed. The correlation test revealed a strong correlation (*r* = 0.973) and a statistically significant result (*p* < 0.001), indicating that the search query was accurate. This method of validation has been utilized in previously published bibliometric investigations [58, 63]. The authors' approach to ensure the accuracy of the search query was meticulous and exhaustive. The participation of two bibliometric experts lent credibility to the findings, and the use of a correlation test provided additional validation. Overall, the authors’ efforts have improved the quality and reliability of the investigation and the research findings.

### Data export and data management

After putting the search strategy into action, the retrieved data were exported to Microsoft Excel in "csv" format. Export data contained information about the titles and abstracts of each document, the countries from which the authors originated, and the institutions with which they were affiliated. Additionally, the data contained information on the annual number of publications, document types, funding agencies, citations, and journal names. The analyses carried out as part of this study focused their attention primarily on the percentages and frequencies of publication. In addition, we utilized Microsoft Office Excel to conduct a linear regression analysis to evaluate the publication trend over time. Therefore, only the ten best-ranked measurements were taken into account. Furthermore, the impact factor (IF) for the top ten journals was calculated using the IF for 2021, which Clarivate Analytics provided in the 2022 Journal Citation Report (JCR). Additionally, the *h*-index, often referred to as the Hirsch index, was used as a qualitative measurement tool to evaluate the performance of scientific studies on nutrition and depression.

Furthermore, network maps were created using VOSviewer software version 1.6.8 (Leiden University, Leiden, The Netherlands) to show the network of terms taken from article titles or abstracts and the collaboration between countries. To forecast future research hotspots, VOSviewer was used to create knowledge networks with a scientific foundation that shows how various research fields are progressing. Using co-occurrence analysis in VOSviewer, terms may be grouped into different clusters, each identified by a unique color. Therefore, cluster analysis of research hotspots can be more effective using a co-occurrence network of terms in the title/abstract. This makes it possible to illustrate and detect a developing trend.

## Results

### General description of the retrieved publications

A total of 2171 publications on nutrition and depression were found between 2002 and 2021, namely 1855 (85.44%) original articles, 190 (8.75%) reviews, 38 (1.75%) letters, and 88 (4.05%) other types of publications.

### Analysis of publication trends

**Fig. 1:**
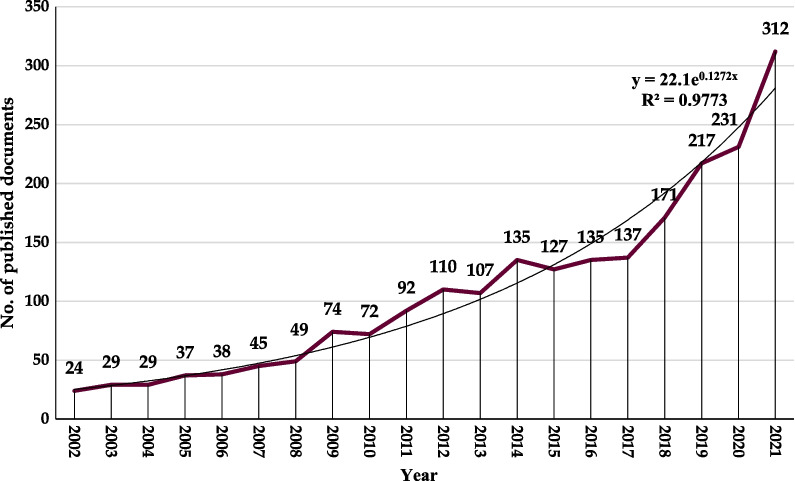
Trends in annual publications on research related to nutrition and depression

The annual number of publications worldwide is shown in Fig. 1. In general, the number of publications rose steadily over time. The early stage (2002–2013) had an average publication of 58.8, showing steady growth. Since 2017, there has been a significant rise in the number of pertinent publications, with 2021 recording the highest amount of research on the connection between nutrition and depression, with 312 papers published. The number of papers published in 2021 was over 13 times greater than that in 2002. As per this study, the linear regression analysis revealed a positive correlation between the number of publications per year and the year of publication (*R*^2^ = 0.977, *p* < 0.001).

### Analysis of countries

One hundred eighteen countries contributed to scientific research on nutrition and depression. In this study, it was found that the USA (*n* = 726; 33.44%) was the most productive country, followed by Australia *(n* = 172; 7.92%), the United Kingdom (*n* = 158; 7.28%), China (*n* = 132; 6.08%), and Canada (*n* = 131; 6.03%) (Table 1). From 2002 to 2021, Fig. 2 depicts the yearly progression of article counts, showing the USA as the leading country in annual publications. Figure 3 is a network visualization map of research collaboration between countries (*n* = 23) with a minimum contribution of 20 articles. As shown by the connecting line's thickness and the nodes' size, the USA had the strongest cross-country collaboration.

**Table 1:** Top 10 countries with the highest publications on research related to nutrition and depression

Ranking	Country	Number of documents	%
1st	USA	726	33.44
2nd	Australia	172	7.92
3rd	United Kingdom	158	7.28
4th	China	132	6.08
5th	Canada	131	6.03
6th	South Korea	128	5.90
7th	Spain	110	5.07
8th	Japan	102	4.70
9th	Iran	90	4.15
10th	Germany	87	4.01

**Fig. 2:**
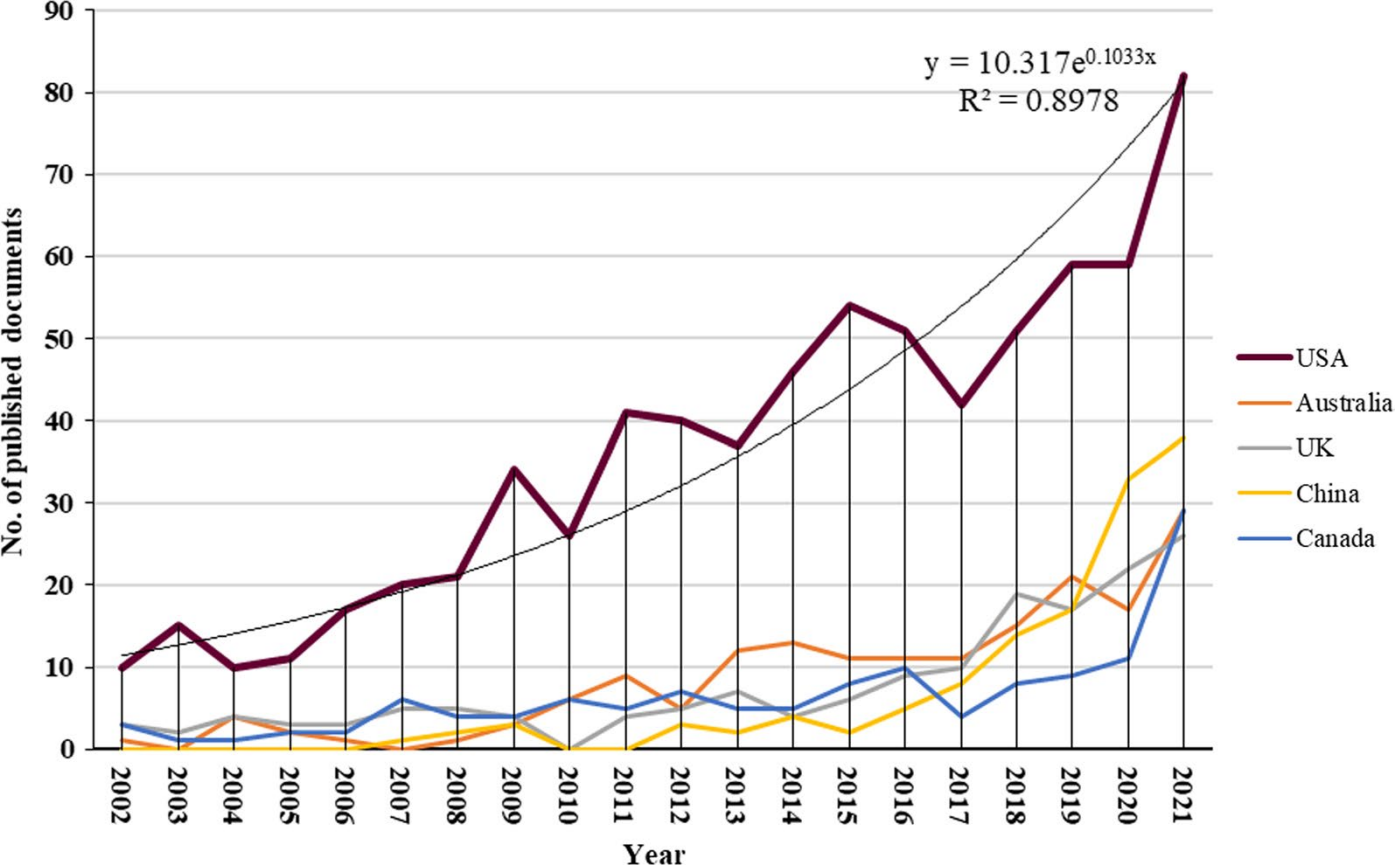
The annual number of publications on research related to nutrition and depression from the top 5 countries between 2002 and 2021

**Fig. 3:**
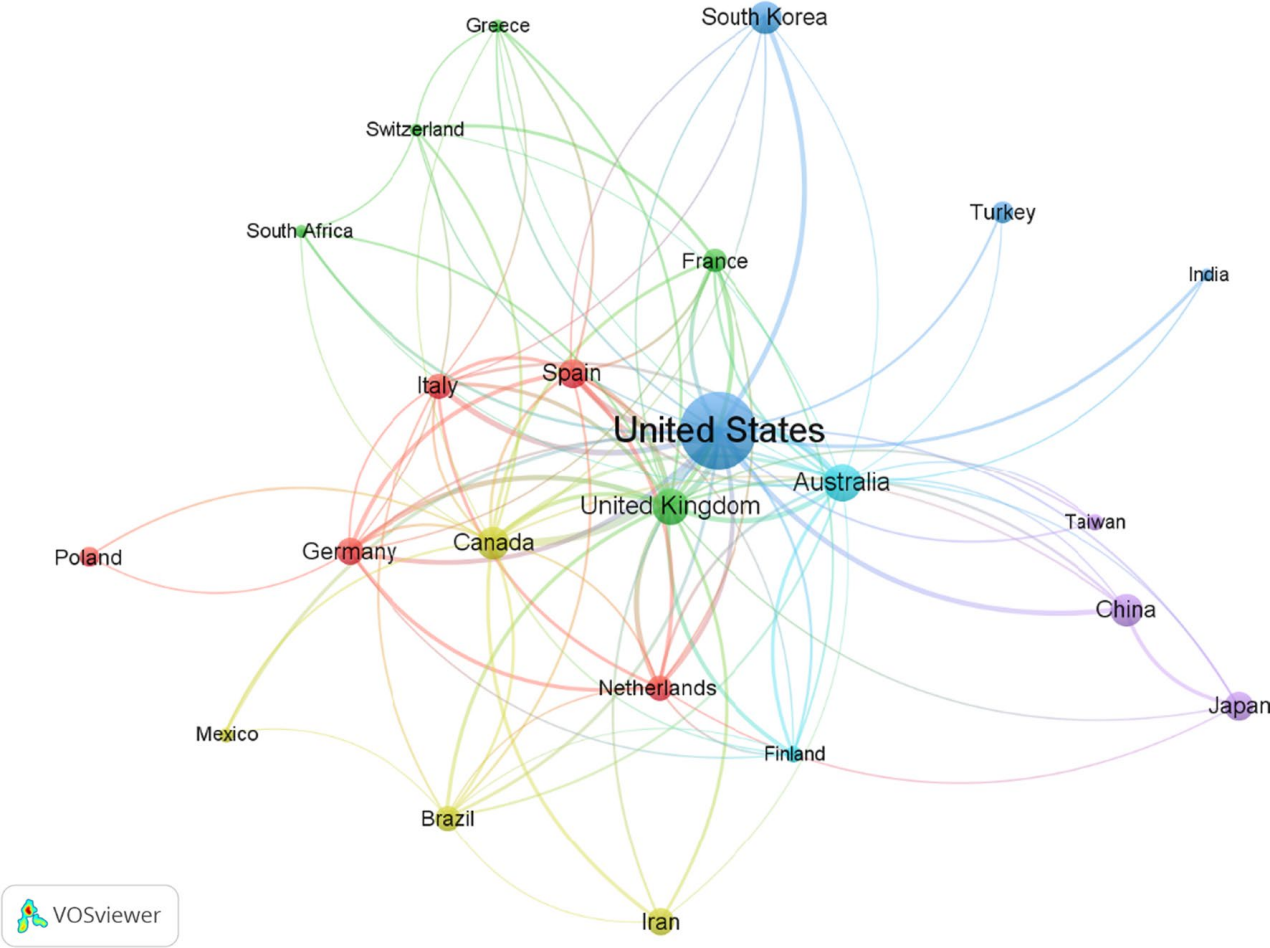
Visualization map of the international research collaboration network with a minimum contribution of 20 documents *per* country set as a threshold (*n* = 23). Countries with short distances and dense connections engage in extensive scientific collaboration. Countries in the periphery with tenuous ties to countries in the center engage in poor international research collaboration. The map was created using VOSviewer software version 1.6.18

### Analysis of institutions

In total, 6,557 institutions have worked in this area. The highest percentage of articles (n = 59, 2.72%) came from *Deakin University*. It was followed by the *University of Melbourne* (*n* = 42, 1.93%), *Tehran University of Medical Sciences* (*n* = 36, 1.66%), and *Harvard Medical School* (*n* = 35, 1.61%). The top ten core institutions that published the most documents on nutrition and depression are listed in Table 2.

**Table 2:** Top 10 institutions with the highest publications on research related to nutrition and depression

Ranking	Institution	Country	*n*	%
1st	*Deakin University*	Australia	59	2.72
2nd	*University of Melbourne*	Australia	42	1.93
3rd	*Tehran University of Medical Sciences*	Iran	36	1.66
4th	*Harvard Medical School*	USA	35	1.61
5th	*Universiteit van Amsterdam*	Netherlands	33	1.52
5th	*Vrije Universiteit Amsterdam*	Netherlands	33	1.52
7th	*INSERM*	France	30	1.38
8th	*School of Nutritional Sciences and Dietetics*	Iran	29	1.34
9th	*King's College London*	UK	28	1.29
10th	*University of Michigan, Ann Arbor*	USA	25	1.15
10th	*Amsterdam Public Health*	Netherlands	25	1.15
10th	*Université Paris Cité*	France	25	1.15

### Analysis of funding agencies

Table 3 presents the top ten funding agencies with the most publications on nutrition and depression. Globally, the *National Institutes of Health* (USA) funded the most articles with the highest number of citations (*n* = 160; 7.37%). The *National Institute of Mental Health* (USA) ranked second (*n* = 98; 4.51%), followed by the *National Institute of Diabetes and Digestive and Kidney Diseases* (USA) (*n* = 57; 2.63%), and the *U.S. Department of Health and Human Services* (USA) (*n* = 55; 2.53%). Seven funding institutions in the USA supported publications on nutrition and depression, while the remaining funding organizations were the *National Natural Science Foundation of China* in China, the *National Health and Medical Research Council* in Australia, and the *Japan Society for the Promotion of Science* in Japan.

**Table 3:** Top 10 funding agencies with the highest publications on research related to nutrition and depression

Ranking	Funding agencies	Country	*n*	%
1st	*National Institutes of Health*	USA	160	7.37
2nd	*National Institute of Mental Health*	USA	98	4.51
3rd	*National Institute of Diabetes and Digestive and Kidney Diseases*	USA	57	2.63
4th	*U.S. Department of Health and Human Services*	USA	55	2.53
5th	*National Health and Medical Research Council*	Australia	53	2.44
6th	*Japan Society for the Promotion of Science*	Japan	43	1.98
7th	*National Natural Science Foundation of China*	China	41	1.89
8th	*National Heart, Lung, and Blood Institute*	USA	37	1.70
9th	*Eunice Kennedy Shriver National Institute of Child Health and Human Development*	USA	36	1.66
10th	*National Institute on Aging*	USA	35	1.61

### Journal analysis

Table 4 lists the top ten journals in this field by productivity. A total of 375 documents were published by the top 10 journals or 17.27% of the total. Most documents were published in the *Journal of Affective Disorders* (n = 88). With 46 publications, *Nutrients* was the second most productive journal. *Eating behaviors* came in third (n = 41) and were followed by public health nutrition (n = 32) and the *British Journal of Nutrition* (*n* = 31).

**Table 4:** Top 10 journals with the highest publications on research related to nutrition and depression

Ranking	Journal	*n*	%	IF^1^
1st	*Journal of Affective Disorders*	88	4.05	6.533
2nd	*Nutrients*	46	2.12	6.706
3rd	*Eating Behaviors*	41	1.89	2.936
4th	*Public Health Nutrition*	32	1.47	4.539
5th	*British Journal of Nutrition*	31	1.43	4.125
6th	*Journal of Dairy Science*	30	1.38	4.225
7th	*Appetite*	28	1.29	5.016
8th	*Psychiatry Research*	28	1.29	11.225
9th	*Nutritional Neuroscience*	26	1.20	4.062
10th	*Eating and Weight Disorders*	25	1.15	3.008

^1^Impact factor (IF) from Journal Citation Reports (Source Clarivate, 2022)

### Analysis of Citations

According to the citation analysis, the retrieved articles have been cited an average of 26.6 times and had an *h*-index of 105 with 57,781 citations. The range of citations was between 0 and 592. Two hundred thirty-nine of the retrieved articles had no citations, while 117 received 100 or more citations. The top ten most-cited articles received a total of 4,686 citations [20, 64–72]. The total number of citations for these articles on nutrition and depression ranged between 404 and 592 (Table 5).

**Table 5:** List of the top 10 cited articles for studies related to nutrition and depression

Ranking	Authors	Title	Year	Source title	Cited by
1st	Onyike et al. [67]	“Is Obesity Associated with Major Depression? Results from the Third National Health and Nutrition Examination Survey”	2003	*American Journal of Epidemiology*	592
2nd	Whitaker et al. [72]	“Food insecurity and the risks of depression and anxiety in mothers and behavior problems in their preschool-aged children”	2006	*Pediatrics*	524
3rd	Rahman et al. [69]	“Impact of maternal depression on infant nutritional status and illness: A cohort study”	2004	*Archives of General Psychiatry*	513
4th	Psaltopoulou et al. [68]	“Mediterranean diet, stroke, cognitive impairment, and depression: A meta-analysis”	2013	*Annals of Neurology*	489
5th	Jacka et al. [65]	“Association of western and traditional diets with depression and anxiety in women”	2010	*American Journal of Psychiatry*	480
6th	Lai et al. [20]	“A systematic review and meta-analysis of dietary patterns and depression in community-dwelling adults”	2014	*American Journal of Clinical Nutrition*	434
7th	Ford and Erlinger [64]	“Depression and C-Reactive Protein in US Adults: Data from the Third National Health and Nutrition Examination Survey”	2004	*Archives of Internal Medicine*	430
8th	Sánchez-Villegas et al. [71]	“Association of the Mediterranean dietary pattern with the incidence of depression: The Seguimiento Universidad de Navarra/University of Navarra follow-up (SUN) cohort”	2009	*Archives of General Psychiatry*	410
9th	Riolo et al. [70]	“Prevalence of depression by race/ethnicity: Findings from the national health and nutrition examination survey III”	2005	*American Journal of Public Health*	410
10th	Olivardia et al. [66]	“Biceps and body image: The relationship between muscularity and self-esteem, depression, and eating disorder symptoms”	2004	*Psychology of Men and Masculinity*	404

### Co-occurrence analysis

**Fig. 4:**
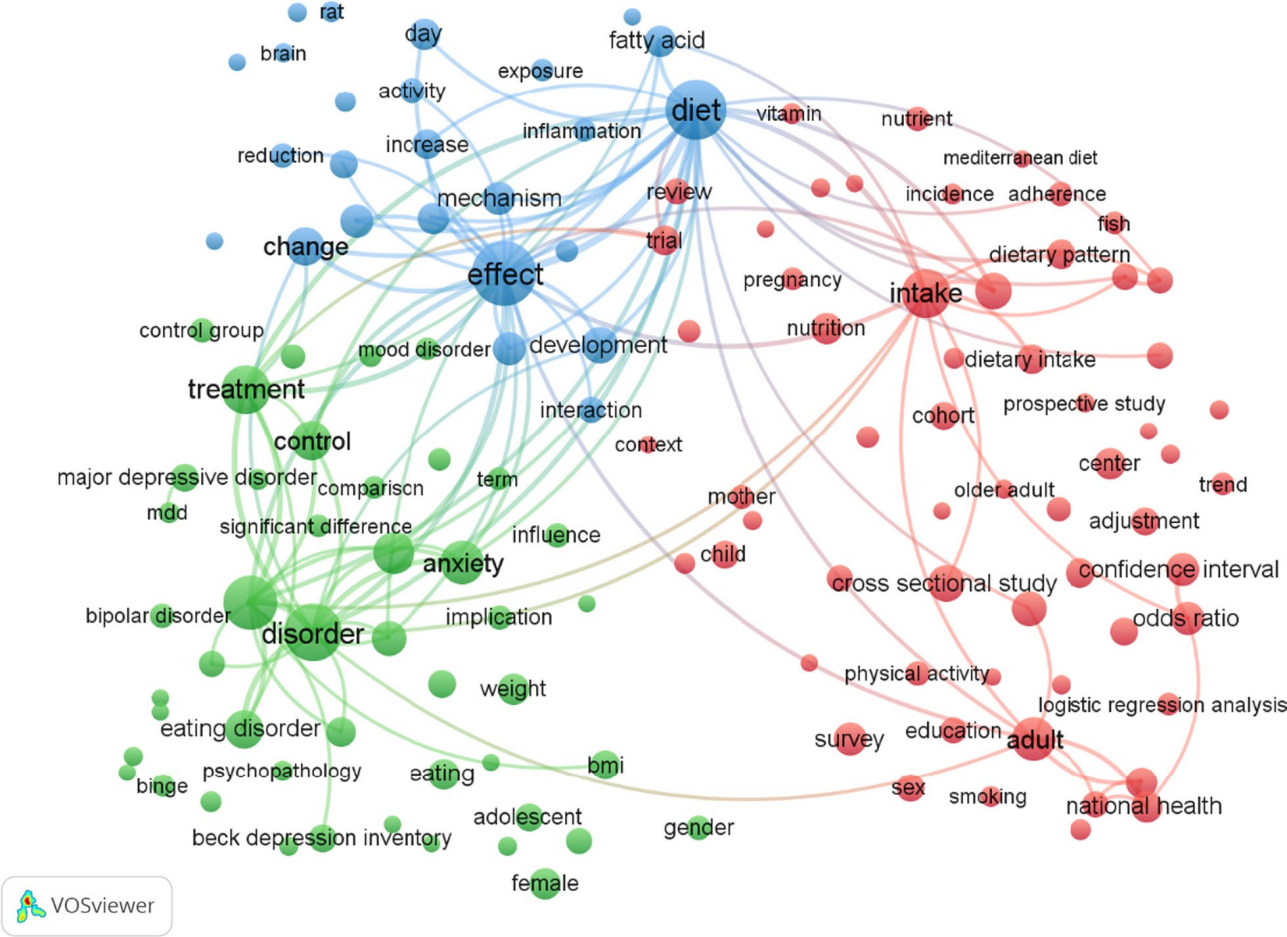
Cluster map based on term analysis appearing in titles or abstracts. The size of the circle indicates occurrences of the terms, and different colors indicate the variety of clusters. The map was created using VOSviewer software version 1.6.18

The co-occurrence network is generated by assessing the number of articles in which terms appeared in titles or abstracts simultaneously. The objective is to identify the hottest research directions and issues essential for monitoring science's growth. As depicted in Fig. 4, 131 detected terms (the minimum number of occurrences of a term in titles and abstracts was greater than 50) were categorized into three clusters. The most frequent terms on the map include those related to (a) fatty acid links to depression and brain inflammation (blue cluster), (b) depression and eating disorders (green cluster), and finally, (c) adherence to the Mediterranean diet and risk of depression (red cluster).

### Future research direction analysis

**Fig. 5:**
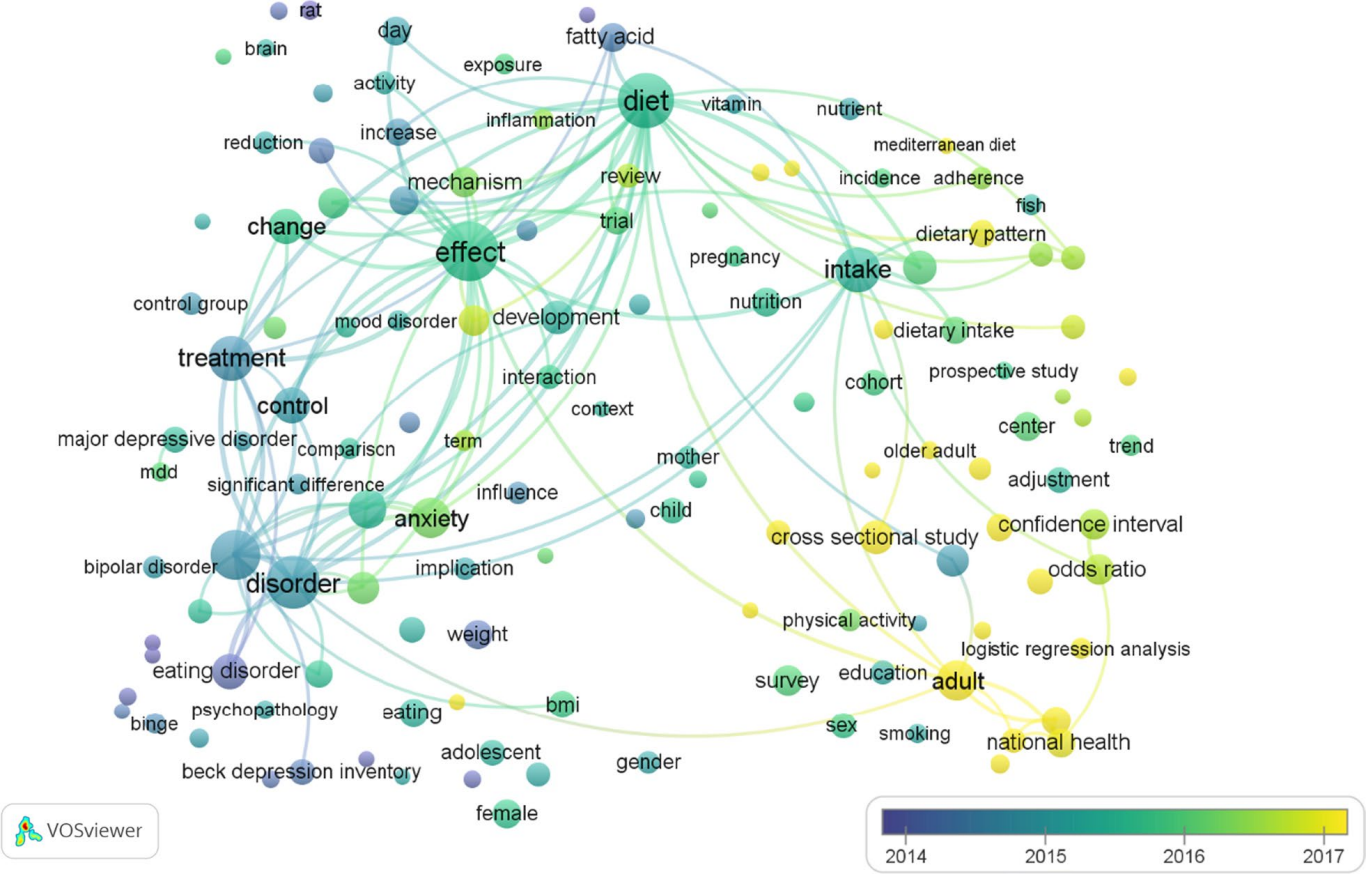
Network visualization map of the analysis of terms in titles according to the frequency of appearance. Blue denotes earlier occurrences of the terms, and yellow denotes later occurrences. The map was created using VOSviewer software version 1.6.18

Figure 5 in VOSviewer depicts each term in a distinct color based on its average frequency across all the publications retrieved. The color yellow corresponds to the terms that appeared most recently, whereas blue signifies the earliest occurrences. Until 2016, the field concentrated mainly on the links between "fatty acids, depression, and brain inflammation," as well as "depression and eating disorders." The research on "the relationship between adherence to the Mediterranean diet and depression risk" is a more recent development, which emerged after 2016.

## Discussion

In this study, we investigated the developmental trends and hotspots of research on nutrition and depression from the Scopus database by performing a bibliometric analysis with the help of VOSviewer software. Specifically, we looked at research published between 2002 and 2021. We were successful in obtaining 2171 documents that were published between 2002 and 2021. The volume of research that assesses the literature related to nutrition and depression has been steadily growing over the past two decades, as revealed in this study. This trend is expected to continue into the foreseeable future.

The results of this study indicate that the western world today has the most influential nations, with the USA taking the lead due to its large number of publications published and citations [73–77]. Australia, however, seems to be doing well based on the pattern of publications in recent years and should seize this trend to carry out more significant research in this field. In addition, our study reveals the lack of studies in lower-middle-income countries (LMICs) and the necessity of different research designs in LMICs to address the issue of depression and nutrition on a global scale.

In general, term clustering and co-occurrence analysis can reveal hot research areas. Three clusters in the network visualization of terms represented the research hotspots throughout the previous 20 years based on the analysis of terms' occurrence frequency. We can determine that the researchers concentrated primarily on the following three aspects by performing a thorough term analysis. The first was adherence to the Mediterranean diet and the risk of depression. In the past 20 years, many studies have emphasized that the Mediterranean diet characterized by a healthy eating pattern of lean protein, complex carbohydrates, olive oil, and a high intake of fruits and vegetables is a protective measure against depression. Furthermore, a Mediterranean diet can boost energy levels, improve mood, and overcome symptoms of depression due to the high content of nutrients such as selenium, folate, and antioxidants. In contrast, the heavily processed ingredients of red meat, refined sugar, and saturated fat increase the risk of depressive behavior and mood disorders [54, 78–83].

The second was fatty acids linked to depression and brain inflammation. Several studies have shown that fatty acids are significantly associated with depression and brain inflammation [84–88]. Diet is known to influence oxidative stress, inflammation, and brain plasticity and function; all of these physiological aspects may play a role in the development of depression [89]. The brain is rich, particularly in long-chain polyunsaturated fatty acids [90]. A cross-sectional study on healthy older adults found that consuming two fish meals a week, especially fatty fish, positively affects neurocognitive functioning [91]. Furthermore, a meta-analysis study found that depressed people demonstrated a clinical benefit from the omega-3 fatty acid dose of eicosapentaenoic acid compared with placebo groups, as they found that people with depressive disorders have a lower level of omega-3 polyunsaturated fatty acids than the healthy control group [92].

In contrast, free fatty acids contribute to obesity, diabetes, and metabolic diseases. Furthermore, recent studies have revealed that fatty acids can significantly increase the prevalence of brain disease and neuropsychiatric disorders [93]. Additionally, a Danish study in 2019 reported that excess body fat of 10 kg could increase the risk of depression by 17% due to biological and psychological effects that result in negative body image and low self-esteem [94]. An unhealthy diet characterized by high saturated fat has a double feature, as fatty acids are a component of a high-calorie diet and act as signaling molecules that can be linked to peripheral metabolic dyshomeostasis and are involved in neuroinflammation and brain disorders [87].

The third was the link between depression and eating disorders. Eating disorder is characterized by a mental disorder in the Diagnostic and Statistical Manual of Mental Disorders, Fifth Edition (DSM-5) that can significantly alter physical health or psychological functioning. More than 50% of people who suffer from an eating disorder will also experience symptoms of depression and vice versa; depression symptoms can lead to a change in appetite and contribute to an eating disorder [95]. A recent study on women aged 15 to 25 found that those with a lifetime diagnosis of major depressive disorder were four times more likely to have a lifetime diagnosis of an eating disorder [96].

In light of the clinical importance of nutrition and depression and the importance of highly cited articles, we performed a qualitative and quantitative analysis of the ten dietary patterns and depression articles that have received the most citations. Our goal was to assist researchers in understanding research quality and trends, better use the classic articles on nutrition and depression, and provide a reference for future research in this field. Additionally, analysis of the most prominent articles helps pinpoint research hotspots in a topic, and the number of citations an article receives indicates its significance in that discipline [97].

## Strengths and limitations

We believe that this study is the first to describe the performance of scientific studies on nutrition and depression using bibliometrics. As a result, researchers might use visual analysis to better the research subjects, research hotspots, and development trends in nutrition and depression. However, it is important to address some limitations. First, we use the Scopus database to search only pertinent articles. Despite the fact that it is the most comprehensive and reliable data source for many academic subjects [98–102], it could also result in the exclusion of publications from other sources such as PubMed and Web of Science. Second, in the current study, a complete list of keywords was used based on those found in previous literature reviews [20, 54–57]. Nevertheless, there is a minor possibility that some keywords were overlooked, resulting in false-negative results. Third, we chose the ten papers with the most citations. However, because citation searches are “time dependent,” older papers are more likely to be cited, and some older pieces may predominate the most recent list of highly cited articles. Fourth, the scope of the present investigation was restricted to the title search and included only nutritional and depression-related search phrases. Therefore, there is a possibility that this analysis missed certain articles that utilized the terms "diet" and "depression" and other related terms as keywords or contained those terms anywhere within the publication's text. Finally, the nature and data encompassed in Scopus are reflected in the results it produces. However, this can cause the research output of active institutions with multiple Scopus profiles to be scattered, resulting in their name being absent from the active list. Similarly, when different names are used to identify a funding agency in various published papers, the same issue arises. To avoid bias, it is necessary to limit the analysis of data concerning the most active institutions and funding agencies to the results obtained from Scopus through the specified method without any manipulations or merging in Scopus output.

## Conclusion

The first-of-its-kind study analyzed global research on nutrition and depression, identifying top themes, countries, and research clusters. The results showed a significant increase in publications in this field, with the USA leading the way. The Mediterranean diet's link to depression was identified as a hot topic, reflecting recent scientific advances. This study demonstrates how bibliometrics can quickly highlight research hotspots and guide future research. The findings offer a practical and instructive reference for anyone interested in this field.

## Data Availability

All data generated or analyzed during this study are included in this published article. In addition, other data sets used during the current study are available from the corresponding authors on reasonable request.

## Abbreviations

PUFAs: Polyunsaturated fatty acids
LMICs: Lower-middle-income countries
DSM-5: Diagnostic and statistical manual of mental disorders
